# Parental work hours and household income as determinants of unhealthy food and beverage intake in young Australian children

**DOI:** 10.1017/S1368980022000349

**Published:** 2022-08

**Authors:** Chelsea E Mauch, Thomas P Wycherley, Lucinda K Bell, Rachel A Laws, Rebecca Byrne, Rebecca K Golley

**Affiliations:** 1 Caring Futures Institute, College of Nursing and Health Sciences, Level 7, SAHMRI building, North Terrace, Flinders University, Adelaide, SA 5000, Australia; 2 Early Prevention of Obesity in Childhood Centre of Research Excellence, Sydney, Australia; 3 Alliance for Research in Exercise, Nutrition and Activity, Allied Health and Human Performance, University of South Australia, Adelaide, Australia; 4 Institute for Physical Activity and Nutrition, School of Exercise and Nutrition Science, Deakin University, Melbourne, Australia; 5 Queensland University of Technology, School of Exercise and Nutrition Sciences, Faculty of Health, Centre for Children’s Health Research, South Brisbane, Australia

**Keywords:** Dietary intake, Children, Work hours, Income, Social determinants

## Abstract

**Objective:**

This study examined parental work hours and household income as determinants of discretionary (energy-dense, nutrient-poor) food and beverage intake in young children, including differences by eating occasion.

**Design:**

Secondary analysis of cross-sectional data. Three hierarchical regression models were conducted with percentage of energy from discretionary food and beverages across the day, at main meals and at snack times being the outcomes. Dietary intake was assessed by 1 × 24-h recall and 1–2 × 24-h food record(s). Both maternal and paternal work hours were included, along with total household income. Covariates included household, parent and child factors.

**Setting:**

Data from the NOURISH/South Australian Infants Dietary Intake studies were collected between 2008 and 2013.

**Participants:**

Participants included 526 mother–child dyads (median (interquartile range) child age 1·99 (1·96, 2·03) years). Forty-one percentage of mothers did not work while 57 % of fathers worked 35–40 h/week. Most (85 %) households had an income of ≥$50 k AUD/year.

**Results:**

Household income was consistently inversely associated with discretionary energy intake (*β* = –0·12 to –0·15). Maternal part-time employment (21–35 h/week) predicted child consumption of discretionary energy at main meals (*β* = 0·10, *P* = 0·04). Paternal unemployment predicted a lower proportion of discretionary energy at snacks (*β* = -0·09, *P* = 0·047).

**Conclusions:**

This work suggests that household income should be addressed as a key opportunity-related barrier to healthy food provision in families of young children. Strategies to reduce the time burden of healthy main meal provision may be required in families where mothers juggle longer part-time working hours with caregiving and domestic duties. The need to consider the role of fathers and other parents/caregivers in shaping children’s intake was also highlighted.

Excess intakes of energy-dense, nutrient-poor foods and beverages are typical of modern, global dietary patterns^(1–3)^. This is contributing to the high rates of obesity and non-communicable diseases such as CVD and diabetes^(4)^. Termed ‘discretionary’ foods and beverages in Australia^(5)^, the mean intake of energy-dense, nutrient-poor foods and beverages amongst adults was more than twice the recommended maximum daily serves in 2011–2012^(6)^. Similarly, the 2001–2003 National Health and Nutrition Examination Survey data from the USA found that 96 % of adults consumed excessive energy from solid fats, added sugars and alcohol^(2)^. Furthermore, the consumption of excess discretionary foods and beverages begins from as early as the second year of life^(1,2,7)^ and increases over time^(7)^. As the early years are critical in the development of food preferences^(8)^, addressing the early intake of discretionary foods and beverages may prevent unhealthy dietary intake in adulthood.

The determinants of dietary intake are broad, including factors at the individual, household and community levels^(9,10)^. Figure 1 outlines child, parent and household-level factors and their proposed relationship with children’s intake of discretionary foods and beverages and was adapted from similar models of the home and family food environment^(10,11)^. Some of these relationships are well supported by research in young children, such as the role of child eating behaviours^(12,13)^ and parental feeding practices^(14)^, while less is understood about the role of family or household-level factors as determinants of young children’s discretionary food and beverage intake. Innate food preferences and child eating behaviours play a key role in children’s acceptance of food and beverages^(8,15)^. However, child preferences and behaviours are set amongst, and shaped by, the home food environment, which includes the resources, structures and behaviours leading to the availability and provision of food to children^(10)^. Parental and household factors such as parental intake and the availability and accessibility of food are strong, consistent determinants of child dietary intake^(16)^. Therefore, parents are commonly targeted as key agents of change in interventions addressing the dietary intake of young children^(17)^.

**Fig. 1:**
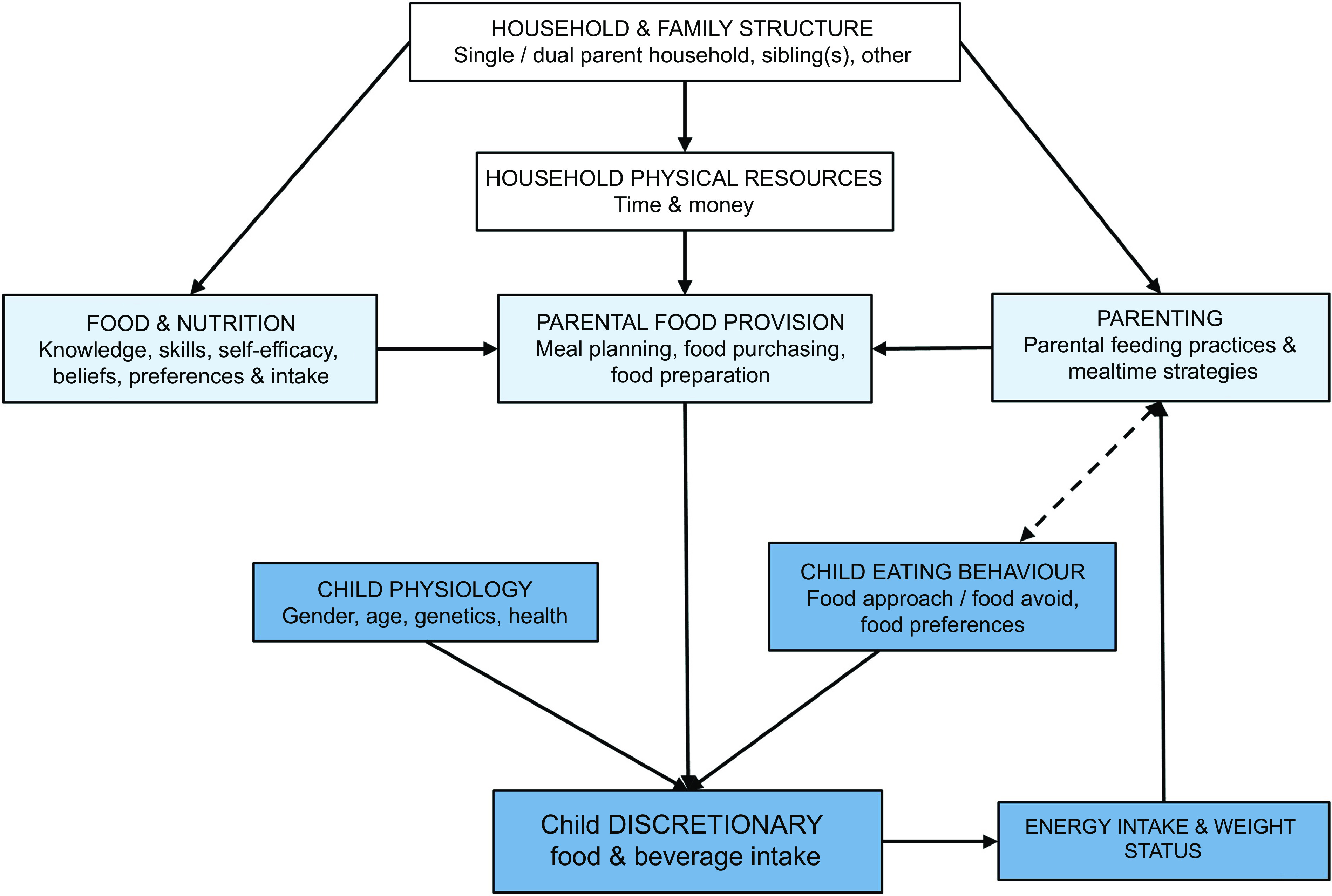
Conceptual model of determinants of young children’s discretionary food and beverage intake, with child factors in dark blue, parent factors in light blue and external family/household factors in white

According to the COM-B framework of behaviour, a combination of capability (C), opportunity (O) and motivation (M) is required in order to perform a behaviour^(18)^. For example, the behaviour ‘parental food provision’ requires nutrition and food knowledge, and cooking skills (capability), a desire, intention or habits that facilitate healthy food provision (motivation) and adequate time, money and other resources to plan, purchase and prepare healthy food and meals (opportunity). Interventions to reduce children’s discretionary food and beverage intake have mainly focused on parental capability and motivation, targeting factors such as child eating behaviour, parental feeding practices, nutrition knowledge and cooking skills, and self-efficacy^(19–23)^. The impact of interventions to reduce young children’s intake of discretionary foods and beverages has so far been modest, suggesting a need to expand this focus to encompass opportunity-related determinants of young children’s dietary intake^(21)^.

Time and money are important determinants of healthy dietary intake in adults^(24)^. Research in school-age children provides evidence of the role of these determinants with respect to discretionary food and beverage intake. A cross-sectional study with 9–13-year-old Australian children found that attitudes, self-efficacy, parental feeding practices and home food availability were important determinants of discretionary food and beverage intake^(25)^. Markers of socio-economic position, such as maternal education, income and employment, moderated the relationship between determinants and intake. Furthermore, the amount of time mothers spent in employment seemed to be more important than maternal occupation^(25)^. These findings suggest that parental work hours and household income play an important role in the discretionary food and beverage intake of school-age children. Their role as determinants of young children’s intake is yet to be investigated however and may be different due to variations in parental influence on child dietary intake over the life course^(26)^.

The time and money required for the provision of food may not be consistent across eating occasions. In Australia, the typical eating pattern consists of three main meals, namely breakfast, lunch and the evening meal, and in young children, close to three snacks per d^(27)^. The food and beverages consumed at these eating occasions differ^(27,28)^. For example, snacks commonly feature ready-to-eat foods such as sweet biscuits and salty snacks, which require minimal preparation^(27,28)^, whereas the evening meal which traditionally incorporates meat, vegetables and grains^(28)^ may take more time to plan, purchase and prepare. Qualitative evidence suggests that main meals are more time intensive to provide, with low-income employed parents citing time scarcity as a key barrier to healthy evening meal provision^(29,30)^. The purchase of fast food after a day at work has been described as a response to time scarcity^(29)^, whereas in a discrete choice experiment, time and money were not found to be important to parents in the selection of snacks for their 3–7-year-old children compared with the influence of child resistance and co-parent support^(31)^. Sub-group analyses found that cost was more important to parents living in lower socio-economic areas, compared with those living in higher socio-economic areas^(31)^. Differences in the determinants of intake across eating occasions would warrant the tailoring of intervention strategies to meal type, although quantitative evidence is required to support this.

This study aimed to examine parental work hours and household income as determinants of discretionary food and beverage intake in young children, and whether their influence differs according to eating occasion. This study will provide evidence regarding opportunity-related determinants of young children’s intake of discretionary foods and beverages, supporting the development of intervention strategies covering all aspects of the COM-B framework^(18)^. Finally, the exploration of the role of parental work hours and household income on young children’s discretionary food and beverage intake at different eating occasions will support more targeted and potentially effective intervention strategies.

## Methods

### Study design, setting and sample

This study was a secondary analysis of cross-sectional data collected as part of the NOURISH and South Australian Infants Dietary Intake studies, conducted in South Australia and Queensland, Australia, between 2008 and 2013. The Strengthening the Reporting of Observational Studies in Epidemiology-Nutritional Epidemiology (STROBE-nut) statement guides study reporting^(32)^. Recruitment and data collection procedures for both studies have been described in detail previously^(22,33)^. Participants included mother–child dyads, where the infant was born at term with no condition or abnormality affecting development or feeding behaviour, and the mother had facility with the English language. NOURISH participants were randomised to receive a feeding intervention promoting positive infant and toddler feeding practices and the development of healthy food preferences, or usual care. Both NOURISH intervention and control participants were included in the present work. South Australian Infants Dietary Intake participants were recruited at the same time and methodology as per the NOURISH control arm. Both cohorts were followed up over the course of 5 years at various ages.

### Data collection

Parent-completed surveys were administered to the same cohort at infant birth, 4–6 months and 2 years and covered parent-reported infant feeding and parenting practices, and maternal and family demographics. The parent involved in data collection was the mother, except *n* 1, where maternal data were provided at all time points except 2 years where the father became the primary carer. Data collected at infant birth included child gender, maternal age and parental educational attainment. Infant and maternal anthropometrics were measured by study staff. Maternal BMI (kg/m^2^) was calculated from weight and height data collected at child aged 4–6 months. All other data were collected at child aged 2 years. Child BMI *Z*-score was calculated using child weight and height measured according to a standardised protocol and the WHO Anthro version 3.0.1 and macros programme (Department of Nutrition for Health and Development, World Health Organization)^(34,35)^.

One 24-h recall and two 24-h food records were conducted with the primary caregiver at child aged 2 years for the collection of child dietary intake data^(22)^. Participants with 2–3 d of intake data were included^(36)^. The 24-h recall utilised a standardised three-pass protocol conducted by trained dietitians via telephone. Food recall and record data included time of consumption, a description of the food and the quantity consumed. Where a primary caregiver felt they could not accurately recall their child’s intake on the day prior, the recall was attempted (unannounced) on another day. An additional food record booklet was provided to be used when the child was being cared for by someone other than the primary caregiver.

### Survey data preparation

Multiple-choice questions, including education, household income, other children and marital status, were collapsed to create dichotomous variables (i.e. *university educated v*. *not university educated*, *less than $50 000 v*. *$50 000 or more, single child v*. *multiple child household, partnered v*. *not partnered*). Categories were based on the distribution of the data, whilst ensuring they were meaningful (i.e. University education being considered a high level of educational attainment, and less than $50 k AUD being considered low income for a family at the time)^(37)^. As past research has found non-linear relationships between maternal work hours and children’s weight and weight-related outcomes^(26,38)^, work hours were grouped into categories and dummy coded for analysis. Maternal working hours (paid employment only) were grouped into four categories, including: *not working (reference category), working 1 to <21 h, 21 to <35 h* and *35 h or more* per week. Paternal working hours (paid employment only) were grouped differently to account for differences in the spread of working hours amongst fathers: *not working, working 1–<35 h, 35–40 h (reference category)* and *greater than 40 h*/week.

Thirty-five Child Eating Behaviour Questionnaire items were used to calculate scores for four sub-scales of ‘food approach’ (*food responsiveness, emotional over-eating, enjoyment of food* and *desire to drink*) and four of ‘food avoid’ (*satiety responsiveness, slowness in eating, emotional under-eating* and *food fussiness*) eating behaviours^(15)^. The internal validity and test–retest reliability of these sub-scales have been established in prior research^(15,39)^. S*atiety responsiveness* and *slowness in eating* were combined into a single score as they have been shown to be highly correlated^(39)^. Mean scores were calculated for the remaining sub-scales, with scores between 1 and 5 indicating low to high levels of each eating behaviour.

Items and sub-scales from the Feeding Practices and Structure Questionnaire represented parental feeding practices^(40)^. Predictive validity has been demonstrated against Child Eating Behaviour Questionnaire sub-scales, and internal reliability demonstrated with Cronbach’s *α* values between 0·61 and 0·87^(40)^. Four of the seven sub-scales were included in the present research, namely *reward for behaviour, reward for eating, covert restriction and overt restriction,* along with a single item to assess *family meal setting*^(40)^.

Nine (1·7 % of 544) participants had missing data on five or more variables and another nine were missing data that were not at random (1·7 % of 544); all eighteen were therefore excluded from the regression analyses. Of the remaining participants (*n* 526), six (1·1 %) had missing data for two variables and sixty-two (11·8 %) for one, which were imputed using maximum likelihood estimation. Descriptive statistics were undertaken on the original sample of 544 (with missing data) and on the sample of 526 with imputed data and were found to be similar.

### Dietary intake data preparation

Food intake data were entered into FoodWorks Professional Version 9 (Xyris Software Pty Ltd), using energy and nutrient data from the 2007 AUSNUT database^(41)^. Data were exported into SPSS Version 22 (IBM) and merged with 8-digit food codes from the AUSNUT 2007 database. Discretionary foods and beverages were identified using the Australian Bureau of Statistics discretionary food flag^(42)^. Discretionary foods and beverages are defined as those that are not essential for meeting nutrient requirements and are generally energy dense, higher in saturated fat, added sugars, Na and/or alcohol and low in fibre^(5)^. Data were cleaned according to a standard protocol^(22)^.

Defined time periods were used to categorise food and beverage intake into eating occasions^(27,43)^, with all food and beverages consumed during these time periods representing main meals and snacks. The time periods were constructed by plotting the energy content of eating occasions across the day for the whole sample to observe when peaks in intake occurred. Main meals included foods and beverages consumed between 06.00–08.59 hours (breakfast), 11.30–14.29 hours (lunch) and 17.00–19.59 hours (evening meal), while snacks included all food and beverages consumed outside of these times. Foods or meals with no time of consumption recorded were excluded from analysis (18/544 (3·3 %) participants with dietary data, a mean (sd) of 654 (484) kJ per participant).

### Data analysis

Analyses were conducted in SPSS version 25 (IBM). Total intake of energy (kilojoules) from discretionary foods and beverages, and discretionary energy consumed at main meals and snacks, was calculated separately for each day of intake and averaged across the number of days reported (*n* 2 or 3). Descriptive statistics for socio-demographic and intake data included medians and interquartile range for continuous variables and counts and percentages for categorical data. Three hierarchical regression models were conducted with the proportion of total energy consumed from discretionary foods and beverages, and the proportion consumed at main meals and snacks. Variables (total of twenty-nine) were entered in six steps, starting with variables representing: (1) parental work hours (six variables) and household income, followed by; (2) household factors (relationship status, highest level of paternal education and number of children in the household); (3) maternal factors (highest level of maternal education, maternal age at infant birth, maternal BMI); (4) parental feeding practices (reward for behaviour, reward for eating, covert restriction, overt restriction, same food as rest of family and intervention condition); (5) child factors (child gender, child BMI *Z*-score and child age) and (6) child eating behaviours (food responsiveness, enjoyment of food, satiety responsiveness/slowness in eating, food fussiness, emotional overeating, emotional undereating and desire to drink). Intervention condition was included in the parental feeding practices step, as the intervention focused on directly addressing these practices. The sample size of 526 allowed for eighteen cases per variable, meeting most sample per variable recommendations for regression analyses^(44)^. There was no multicollinearity, assessed by correlations, tolerance values and variance inflation factor values. Unstandardised beta (*β*), standard error for the unstandardised beta (se), standardised beta (*β*) and adjusted *R*^2^ values are presented. Statistical significance was set at *P* ≤ 0·05.

## Results

**Fig. 2:**
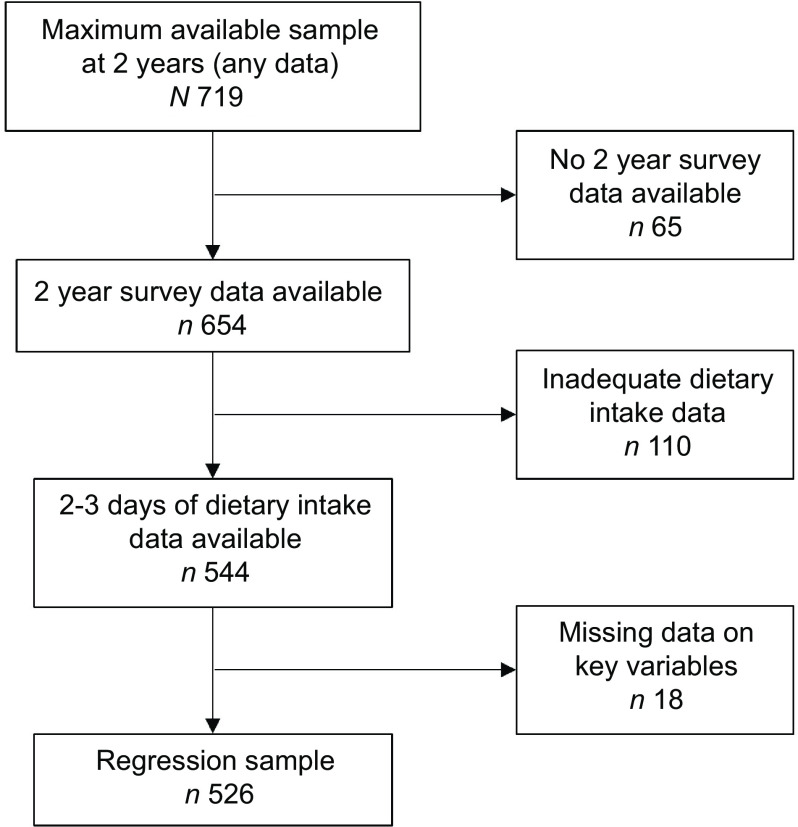
Study participants based on survey and dietary intake data availability

Figure 2 describes the study participants, and Table 1 presents demographic information for the maximum available sample at child aged 2 years. Seven hundred and nineteen participants provided some data at 2 years, with 654 providing survey data. Mothers were mostly partnered (95 %, *n* 618/654) with a household income over $50 000 AUD per year (82 %, *n* 518/631). Just over half were university educated (58 %, *n* 417/716) and less than half (43 %, *n* 275/639) were not working, while fathers were mostly working full time (82 %, *n* 525/640). Participants retained at the 2-year data collection point were older and more likely to hold university qualifications compared with the maximum available sample at baseline (data published elsewhere)^(45)^. Five hundred and forty-four children had 2 (*n* 10) or 3 (*n* 534) days of dietary intake data, of which eighteen were excluded from the regression analyses due to missing data, resulting in a final sample size of 526. Compared with the maximum available sample, the regression sample (*n* 526) included mothers who were slightly older (median (interquartile range) 32 (28–35) years *v*. 31 (28–35) years), more likely to be partnered (*n* 513/526, 98 % *v*. *n* 618/654, 94 %), university educated (325/526, 62 % *v*. 417/716, 58 %) and of a higher income (449/526, 85 % *v*. 518/631, 82 %). Discretionary foods and beverages contributed almost one-fifth of children’s total daily energy intake (19·6 %). Main meals contributed a larger overall proportion of energy intake from discretionary foods and beverages than snacks (554 (313–856) kJ compared with 313 (146–522) kJ).

**Table 1:** Child, parental and household characteristics of the maximum sample at child aged 2 years (*n* 719) and regression sample (*n* 526)

Characteristic	Categories	*n*	Maximum available sample*		Regression sample† (*n* 526)	
			*n* or median	% or IQR	*n* or median	% or IQR
Child variables						
Child gender	Male	716	338	47	240	46
	Female		378	53	286	54
Child age (years)		713	2·00	1·97, 2·03	1·99	1·96, 2·03
Child BMI *Z*-score		704	0·76	0·06, 1·47	0·79	0·12, 1·51
Food responsiveness‡		653	2·2	1·8, 2·6	2·2	1·8, 2·6
Enjoyment of food‡		653	4·0	3·5, 4·3	4·0	3·5, 4·3
Satiety responsiveness/slowness in eating‡		653	3·0	2·7, 3·3	3·0	2·7, 3·3
Food fussiness‡		653	2·5	2·0, 2·8	2·5	2·0, 2·8
Emotional overeating‡		652	1·5	1·0, 2·0	1·5	1·0, 2·0
Emotional undereating‡		653	3·0	2·3, 3·5	3·0	2·3, 3·5
Desire to drink‡		653	2·7	2·3, 3·3	2·7	2·3, 3·3
% EI from discretionary food/beverages – total daily			N/A		19·6	13·2, 27·8
% EI from discretionary food/beverages – main meals only			N/A		11·7	6·7, 17·8
% EI from discretionary food/beverages – snacks only			N/A		6·5	2·9, 10·8††
Parental and family variables						
Maternal age (years)§		716	31·0	28·0, 35·0	32·0	28·0, 35·0
Maternal BMI (kg/m^2^)‖		701	24·8	22·2, 28·6	24·6	22·1, 28·2
Marital status	Single	654	36	6	13	2
	Partnered		618	94	513	98
Maternal education§	University	716	417	58	325	62
	No university		299	42	201	38
Paternal education§	University	700	298	43	232	44
	No university		402	57	294	56
Maternal work hours¶	Not working	639	275	43	217	41
	1 to <21 h		166	26	146	28
	21 to <35 h		135	21	115	22
	35+ h		63	10	48	9
Paternal work hours	Not working	640	26	4	20	4
	1 to <35 h		53	8	54	10
	35 to 40 h		360	56	301	57
	>40 h		165	26	138	26
	N/A		36	6	13	2
Household income	Less than 50 k/year	631	113	18	77	15
	50 k/year or more		518	82	449	85
Number of children	One	634	356	56	289	55
	More than one		278	44	237	45
Study allocation	NOURISH intervention	719	253	35	172	33
	NOURISH Control		279	39	187	36
	SAIDI		187	26	167	32
Reward for behaviour**		653	1·5	1·3, 2·3	1·5	1·3, 2·3
Reward for eating**		654	1·5	1·0, 2·3	1·5	1·0, 2·0
Covert restriction**		652	3·3	2·5, 3·8	3·3	2·5, 3·8
Overt restriction**		653	3·5	2·8, 4·0	3·5	2·8, 4·0
Same food**		652	5·0	3·0, 5·0	5·0	3·0, 5·0

IQR, interquartile range; EI, energy intake; N/A, dietary data not available; SAIDI, South Australian Infants Dietary Intake.

* Sample size varies between *n* 631 and *n* 719 due to missing data, with *n* 719 providing some data at 2-year data collection, of which 654 provided survey data.

† Regression sample includes imputed missing data.

‡ Child Eating Behaviour Questionnaire^(15)^ sub-scales – scores from 1 to 5, with higher scores indicating more of the behaviour.

§ Data collected at recruitment/child birth.

‖ Data collected at time 1/child aged 4–6 months.

¶ Includes *n* 1 father, who became primary carer before T3 measurements (all other maternal data are from the mother at earlier time points).

** Food Parenting and Structure Questionnaire^(40)^ sub-scales/items – score between 1 and 5, with higher scores indicating more of the parenting practice.

†† *n* 525 participants as one child was considered a ‘non-consumer’ of snacks.

The final regression models (after all six steps) investigating the relationship between parental work hours, household income and children’s discretionary food and beverage intake are presented in Table 2. Household income showed a consistent, inverse relationship with children’s discretionary food and beverage intake across all three models (*β* = –0·15, *P* = 0·002; *β* = –0·12, *P* = 0·02 and *β* = –0·13, *P* = 0·01 for total discretionary energy intake at main meals and snacks combined, at main meals only and at snacks only, respectively). Children of families with a gross household income below $50 000 AUD/year consumed significantly more energy from discretionary foods and beverages (irrespective of eating occasion) than those with household incomes of $50 000 AUD/year or more.

**Table 2:** Regression analyses of parental work hours and household income, family, parent and child factors, and proportion of total energy intake from discretionary foods and beverages and at main meals and snacks, in 2-year-old Australian children

Step†		Variable	% Daily energy from discretionary food/bevs (*n* 526)			% Daily energy from discretionary food/bevs consumed at main meals (*n* 526)			% Daily energy from discretionary food/bevs consumed at snacks (*n* 525)‡		
			*β*	se	*β*	*β*	se	*β*	*β*	se	*β*
1	Parental work hours and household income	Maternal working hours (ref: not working)									
		1 to <21 h	0·86	1·14	0·04	0·19	0·86	0·01	0·83	0·65	0·06
		21 to <35 h	2·81	1·29	0·11*	2·04	0·98	0·10*	0·67	0·74	0·05
		35+ h	−0·74	1·74	−0·02	−0·16	1·33	−0·01	−0·53	1·00	−0·03
		Paternal working hours (ref: 35 to 40 h)									
		Not working	−1·74	2·49	−0·03	0·69	1·90	0·02	−2·84	1·43	−0·09*
		1 to <35 h	−0·56	1·60	−0·02	−1·12	1·21	−0·04	−0·01	0·91	−0·00
		>40 h	−1·96	1·07	−0·08	−2·11	0·81	−0·11*	−0·26	0·61	−0·02
		Household income (ref: <50 k)	−4·60	1·47	−0·15**	−2·69	1·12	−0·12*	−2·16	0·84	−0·13*
2	Household factors	Partnered (ref: single)	3·15	3·14	0·05	3·33	2·39	0·06	−0·00	1·79	0·00
		Paternal education (ref: no university)§	−0·82	1·01	−0·04	−1·93	0·77	−0·12*	1·44	0·58	0·12*
		No of children (ref: one child)	1·11	1·03	0·05	1·22	0·79	0·07	0·08	0·59	0·01
3	Maternal factors	Maternal education (ref: no university)§	−1·41	1·05	−0·06	−1·10	0·80	−0·07	−0·41	0·60	−0·03
		Maternal age§	−0·07	0·10	−0·03	−0·06	0·08	−0·03	−0·04	0·06	−0·03
		Maternal BMI‖	0·06	0·09	0·03	0·06	0·07	0·04	0·02	0·05	0·02
4	Parental feeding practices	Reward for behaviour	1·63	0·85	0·10	0·82	0·65	0·07	0·73	0·49	0·08
		Reward for eating	1·77	0·77	0·12*	1·35	0·58	0·12*	0·28	0·44	0·04
		Covert restriction	−2·00	0·54	−0·16***	−1·33	0·41	−0·14**	−0·94	0·31	−0·14**
		Overt restriction	−0·49	0·56	−0·04	−0·31	0·43	−0·03	−0·34	0·32	−0·05
		Same food	0·06	0·44	0·01	−0·41	0·33	−0·06	0·44	0·25	0·09
		Group allocation (ref: NOURISH control/SAIDI)	−1·30	1·04	−0·06	−1·03	0·79	−0·06	−0·81	0·59	−0·06
5	Child factors	Child gender (ref: male)	1·10	0·92	0·05	0·40	0·70	0·02	0·68	0·53	0·06
		Child age	−1·76	8·29	−0·01	1·57	6·30	0·01	−3·21	4·74	−0·03
		Child BMI *Z*-score	−0·72	0·47	−0·07	−0·70	0·36	−0·09	−0·18	0·27	−0·03
6	Child eating behaviours	Food responsiveness	−0·38	0·96	−0·02	−0·14	0·73	−0·01	−0·20	0·55	−0·02
		Enjoyment of food	0·09	1·14	0·01	0·18	0·86	0·01	0·30	0·65	0·03
		Satiety and slowness	2·47	1·11	0·12*	1·82	0·84	0·12*	0·67	0·63	0·06
		Food fussiness	−0·24	0·93	−0·02	−0·70	0·70	−0·06	0·81	0·53	0·09
		Emotional overeating	1·75	1·13	0·08	0·99	0·86	0·06	0·68	0·65	0·06
		Emotional undereating	−1·11	0·58	−0·09	−1·01	0·44	−0·11*	0·16	0·34	0·02
		Desire to drink	−0·16	0·58	−0·01	−0·17	0·44	−0·02	−0·03	0·33	−0·00
Adjusted *R*^2^				0·117***			0·114***			0·052**	

Ref, reference category; SAIDI, South Australian Infants Dietary Intake.

* *P* < 0 05.

† Only the final results of the hierarchical models are displayed.

‡ *n* 1 participant excluded as they were considered a non-consumer of snacks.

§ At recruitment/child birth.

‖ At Time 1/child aged 4–6 months.

** *P* < 0 01.

*** *P* < 0 001.

Maternal work hours contributed significantly to the total and main meal models, after controlling for covariates (see online supplementary material, Supplemental Tables 1 and 2). Children with mothers working 21–35 h/week consumed significantly more total energy from discretionary foods and beverages (*β* = 0·11, *P* = 0·03) and at main meals (*β* = 0·10, *P* = 0·04) than children with mothers who were not working (reference group), whereas children with fathers working greater than 40 h/week had a lower intake of discretionary foods and beverages at main meals (*β* = –0·11, *P* = 0·01), compared with their peers with fathers working a standard full-time week of 35–40 h. This was independent of paternal education and household income, both of which were also inversely associated with discretionary intake at main meals (*β* = –0·12, *P* = 0·01 and *β* = –0·12, *P* = 0·02, respectively).

Although maternal work hours were not associated with the intake of discretionary foods and beverages at snacks, children with fathers not working consumed less discretionary foods and beverages at snacks (*β* = −0·09, *P* = 0·047). The association between snack discretionary intake with paternal education was the opposite of that found for main meals, where children with fathers that had a university education consumed more energy from discretionary foods and beverages at snacks than children with fathers without a university education (*β* = 0·12, *P* = 0·01). Of the remaining covariates, covert restriction was found to be the most important determinant of children’s discretionary food and beverage intake across all three models (*β* = –0·16, *P* < 0·001; *β* = –0·14, *P* = 0·001 and *β* = –0·14, *P* = 0·003, respectively). Children whose mothers reported using more covert restriction practices consumed a lower total proportion of energy from discretionary foods and beverages, and a lower proportion at main meals and snacks.

Overall, the models accounted for 11·7 % of the variance in children’s total discretionary food and beverage intake (*R*^2^ = 0·117, *P* < 0·001), 11·4 % in discretionary food and beverage intake at main meals (*R*^2^ = 0·114, *P* < 0·001) and 5·2 % at snacks (*R*^2^ = 0·052, *P* = 0·002). The majority of variance was accounted for by the parental work hours and household income (Step 1) and primary carer parenting (Step 4) steps of the regressions (see online supplementary material, Supplemental Tables 1–3).

## Discussion

This study explored parental work hours and household income as opportunity-related determinants of discretionary food and beverage intake in young Australian children and investigated differential associations across eating occasions. Household income had a strong, inverse association with children’s discretionary food and beverage intake across all eating occasions. Maternal and paternal work hours were also key determinants of young children’s discretionary food and beverage intake. Maternal work hours had a non-linear relationship with young children’s discretionary food and beverage intake at main meals, with children of mothers working 21–35 h/week consuming more than those of mothers who were not working, whereas children of fathers working more than 40 h/week had a lower intake of discretionary foods and beverages at main meals compared with those working 35–40 h, and children of fathers who were not working consumed less at snacks. These findings suggest that intervention strategies addressing young children’s intake of discretionary foods and beverages should consider opportunity-related determinants of intake such as maternal and paternal time and money and may benefit from tailoring according to eating occasion.

### Household income

Consistent with prior research, household income was inversely associated with children’s discretionary food and beverage intake in all three models^(7,46)^. Children from households with an income of less than $50 000 AUD/year (i.e. the bottom two quintiles for gross household income in Australia in 2015–2016^(37)^) consumed around 4·6 % more energy from discretionary foods and beverages than those from households with an income of $50 000 AUD or more. Both measurable income and ‘feeling poor’ have been associated with dietary intake in adults, with the effect being stronger with persisting scarcity^(24)^. The mechanisms of this relationship are complex, being that there is no substantial difference between the cost of healthy and unhealthy diets^(47)^. The cost of diets in line with the dietary guidelines is between 88 and 99 % of the cost of current, unhealthy diets in Australian families^(47)^. Similarly in some populations in New Zealand, such as those with a higher energy intake, the cost of a healthy diet is lower than that of current diets^(48)^. Furthermore, modelling has shown that even when time cost is taken into account, healthier home-assembled and home-made meals were generally cheaper than takeaway meals^(49)^. However, families with a low disposable income may be driven to serve acceptable foods that are not rejected and wasted, such as palatable and shelf-stable discretionary foods and beverages^(30)^. A low disposable income may also act as a barrier to the purchase of other tools supporting healthy food preparation, such as healthy pre-prepared meals and cooking equipment. Regardless of the mechanism, this research shows that household income is an important opportunity-related determinant of children’s discretionary food and beverage intake and must be considered when planning interventions or policy strategies to address intake.

### Parental work hours

Both maternal and paternal work hours were associated with young children’s intake of energy from discretionary foods and beverages. Children with mothers working 21–35 h/week consumed on average 2·8 % more energy from discretionary foods and beverages daily than children with mothers who worked up to 21 h/week, full time (35+ h/week) or were not working. Research in preschool and school-age children has found that greater maternal work hours are associated with lower dietary quality^(50,51)^. This may be through the impact of work hours on time available for food-related behaviour such as shopping, cooking and eating with children^(52)^. In US school-age children, a 20-h increase in maternal work hours was associated with an increased likelihood of consuming fast food at least once per week and consuming sugar-sweetened beverages at least once per day^(50)^. Similarly in a study of multiple European countries, full-time maternal employment was found to be negatively associated with children’s diet quality, although the effect was relatively small^(51)^. The inclusion of a broad range of parent and child covariates, and the younger age of our sample may account for the difference in findings of the present work.

The inclusion of paternal work hours in this study was unique, with prior research not generally accounting for this factor^(26,38,53)^. Fathers work time has tended to be viewed as unimportant in public policy related to parenting, despite their important role in contributing key family resources such as time and money^(54)^. Children with fathers working greater than 40 h/week consumed less energy in the form of discretionary foods and beverages at main meals, while children of fathers who were not working consumed less at snacks. Although mothers are more frequently the primary caregiver and food provider in Australian households^(55)^, these findings are a reminder that the father or father figure is also a key influencer of young children’s discretionary food and beverage intake^(56)^. Whether this is through their direct contribution to food-related tasks or role modelling, or the provision of support to the primary food provider^(57)^ or through the increased use of external supports such as childcare and/or extended family, the mechanism underlying these findings is unclear and warrants further investigation.

There is no simple explanation for the non-linear findings of this study in relation to maternal and paternal work hours. Past research has similarly demonstrated non-linear relationships between parental work hours and weight and weight-related behaviours^(26,38,53)^. One possible explanation may be that low maternal work hours allow more opportunity for food-related processes, whilst full-time maternal and/or paternal work hours may necessitate a level of organisation and flexibility regarding food-related processes that offer some protection. For example, women working full-time may seek external support with food provision, or outsource other household tasks such as cleaning to allow more time for food provision^(58)^. Furthermore, the enrichment that full-time employment may add to maternal and paternal capability, for example, may outweigh negative effects on time availability for food provision^(26)^. However, these relationships may not be due to the effect of work hours on time available for food provision at all and may not be causal. More research, including qualitative research, is needed to understand if these relationships are due to the availability or scarcity of time, or some other mechanism, such as self-efficacy.

The relationship between children’s discretionary food and beverage intake and parental work hours varied by eating occasion. Both maternal and paternal work hours were associated with children’s intake of discretionary foods and beverages at main meals, whilst only paternal work hours were important at snacks. Main meals require more planning and preparation than snacks; thus, work hours may be a more important determinant of children’s discretionary food and beverage intake at main meals compared with snacks. Horning *et al.*^(59)^, in their work investigating mainly mothers’ reasons for purchasing packaged, processed meals, found that those who worked more hours were more likely to report time scarcity as a reason for purchasing convenience foods. By contrast, a discrete choice experiment with mainly mothers of children aged 3–7 years found that time was not a significant factor influencing parental snack choice when weighed up against child acceptance or resistance, co-parent support and home food availability^(31)^. This suggests that different intervention approaches may be required at different eating occasions, with time constraints possibly being more relevant when targeting main meals.

### Covert restriction

Of the covariates, the parental feeding practices step resulted in the largest increase in variance for all models owing to the parental feeding practice ‘covert restriction’, while child factors such as gender, age, BMI *Z*-score and eating behaviour were less important in this age group. Covert restriction is the act of restricting a child’s food environment so that they are unaware of it; for example, by avoiding the purchase of discretionary foods and beverages^(60)^. This contrasts with overt restriction which includes more direct control and restriction of child intake^(25,60)^. Similar work in children of various ages confirms the importance of covert restriction in limiting discretionary food and beverage intake^(25,61)^. This highlights the importance of targeting parental capability with respect to setting up a healthy home food environment, particularly in families with young children.

### Strengths and limitations

The incorporation of both maternal and paternal factors was a key strength of this work, as it recognises the influence of both parents on children’s dietary intake, whilst the inclusion of a broad range of covariates ensured that key parent and child factors were adjusted for. This research was however limited by the use of work hours as a proxy for time available for food provision, as work hours do not take into account time commitments outside of work and when and where work takes place (e.g. shift work and working from home). Furthermore, this economic perspective of time does not address the perception of time scarcity, which has been identified as equally important as measurable time^(24)^. Similarly, gross household income does not take into account the availability of money for food and food-related purchases after tax and other essential expenses such as mortgage repayments or rent. Nor does it consider self-assessed poorness, which has been shown to be associated with an increased likelihood of consuming energy from discretionary foods and beverages in adults^(24)^.

As with similar community-based obesity prevention studies^(19)^, NOURISH parents were older, of a higher education and more likely to be partnered than the broader population^(22)^. This may explain the lower discretionary food and beverage intake, 20 % in this sample of children compared with 30 % in 2–3-year-olds in the Australian Health Survey^(62)^. The use of parent-reported 24-h food recalls and records was a strength of this work; however, these measures are prone to social desirability bias leading to possible underreporting of children’s discretionary food and beverage intake^(63)^. Finally, the amount of variance explained by the models was relatively small, although of a similar magnitude to a study investigating home environment determinants of intake in school-age children (9 and 16 % for sweet and savoury snacks and high-energy beverages, respectively)^(64)^. Although child, parent and household factors were demonstrated to be important in the present work, there are clearly other determinants at play that were not captured. For example, home food environment factors such as food availability and parent intake^(65)^, local food environment factors such as supermarkets, food outlets and childcare centres, and food-related policy such as those influencing food pricing and marketing^(10)^ were not included or adjusted for in the analysis.

### Recommendations for future research

Future research in this space would benefit from the inclusion of variables that better represent time and income availability and feelings of scarcity in families of young children, and the use of more socio-economically diverse samples. Analyses that allow the investigation of pathways and interactions between maternal and paternal work hours, and work hours and income, may support a deeper understanding of the interplay between time and money. Intervention development should consider strategies that enable behaviour by increasing means or reducing barriers, by restructuring the physical or social environment or by imparting skills, as these are thought to be effective in addressing opportunity-related determinants of behaviour such as time and money^(18)^, although care must be taken to ensure that interventions and programmes are widely accessible and do not increase social inequities between those who can afford or access support and those who cannot^(58)^.

## Conclusion

This investigation of opportunity-related determinants of young children’s discretionary food and beverage intake through a novel eating occasions lens has provided evidence that can be used to enhance future interventions. Parental work hours and household income were found to be key determinants of young children’s discretionary food and beverage intake, along with parental factors such as covert restriction. Household income was a strong and consistent determinant of children’s discretionary food and beverage intake across all eating occasions, meaning that intervention and policy strategies targeting discretionary food and beverage intake in young children should consider the financial implications of dietary change. The maternal work profile of 21–35 h/week was associated with greater child intake of discretionary foods and beverages at main meals, suggesting that this group may require more support to manage the competing demands of work, caregiving and domestic duties, such as evening meal preparation. However, amongst an increasing body of research considering the role of fathers (or father figures) in shaping children’s food intake and preferences^(66,67)^, this study also suggests a need to consider fathers and other parents or caregivers in future dietary interventions.

## Supplementary material

For supplementary material accompanying this paper visit https://doi.org/10.1017/S1368980022000349. click here to view supplementary material
