# Supplementary material for: Parental work hours and household income as determinants of unhealthy food and beverage intake in young Australian children
Source: Public Health Nutr. 2022 Feb 9;25(8):2125–36. doi: 10.1017/S1368980022000349 (PMC9991677; doi:10.1017/S1368980022000349)
Supplement: Supplementary file 1 [file S1368980022000349sup.zip › S1368980022000349sup002.docx]

Supplementary Table 1: Regression analyses of parental work hours and household income, family, parent and child factors, and proportion of total energy intake from discretionary foods and beverages (main meals and snacks combined) in 2-year-old Australian children (n=526)

|  | **Step 1** | | **Step 2** | | **Step 3** | | **Step 4** | | **Step 5** | | **Step 6** | |
| --- | --- | --- | --- | --- | --- | --- | --- | --- | --- | --- | --- | --- |
|  | **B (SE)** | **β** | **B (SE)** | **β** | **B (SE)** | **β** | **B (SE)** | **β** | **B (SE)** | **β** | **B (SE)** | **β** |
| Maternal working hrs (ref: not working) | |  |  |  |  |  |  |  |  |  |  |  |
| 1 to <21 hrs | 0.33 (1.15) | 0.01 | 0.45 (1.16) | 0.02 | 0.78 (1.17) | 0.03 | 0.68 (1.13) | 0.03 | 0.77 (1.13) | 0.03 | 0.86 (1.14) | 0.04 |
| 21 to <35 hrs | 2.21 (1.24) | 0.09 | 2.75 (1.30) | 0.11* | 3.21 (1.31) | 0.12* | 2.34 (1.28) | 0.09 | 2.62 (1.29) | 0.10* | 2.81 (1.29) | 0.11* |
| 35+ hrs | -0.43 (1.74) | -0.01 | -0.06 (1.78) | -0.00 | 0.25 (1.79) | 0.01 | -1.10 (1.75) | -0.03 | -0.89 (1.74) | -0.02 | -0.74 (1.74) | -0.02 |
| Paternal working hrs (ref: 35 to 40 hrs) | |  |  |  |  |  |  |  |  |  |  |  |
| Not working | -3.50 (2.51) | -0.06 | -2.93 (2.54) | -0.05 | -2.61 (2.54) | -0.05 | -1.51 (2.48) | -0.03 | -1.99 (2.49) | -0.04 | -1.74 (2.49) | -0.03 |
| 1 to <35 hrs | -0.54 (1.61) | -0.02 | -1.00 (1.63) | -0.03 | -0.68 (1.63) | -0.02 | -0.23 (1.59) | -0.01 | -0.42 (1.59) | -0.01 | -0.56 (1.60) | -0.02 |
| >40 hrs | -1.27 (1.10) | -0.05 | -1.51 (1.10) | -0.06 | -1.60 (1.10) | -0.07 | -1.79 (1.07) | -0.07 | -1.81 (1.07) | -0.07 | -1.96 (1.07) | -0.08 |
| Household income (ref: <50k) | -5.06 (1.39) | -0.17*** | -4.82 (1.50) | -0.16** | -4.18 (1.51) | -0.14** | -4.35 (1.47) | -0.14** | -4.28 (1.46) | -0.14** | -4.60 (1.47) | -0.15** |
| Partnered (ref: single) |  |  | 1.90 (3.22) | 0.03 | 1.78 (3.21) | 0.03 | 1.67 (3.10) | 0.02 | 2.05 (3.10) | 0.03 | 3.15 (3.14) | 0.05 |
| Paternal education (ref: no uni)^a^ |  |  | -2.09 (0.96) | -0.10* | -1.09 (1.04) | -0.05* | -0.90 (1.01) | -0.04 | -0.79 (1.01) | -0.04 | -0.82 (1.01) | -0.04 |
| No of children (ref: one child) |  |  | 1.35 (1.00) | 0.06 | 1.59 (1.04) | 0.07 | 0.96 (1.03) | 0.04 | 0.89 (1.03) | 0.04 | 1.11 (1.03) | 0.05 |
| Maternal education (ref: no uni)^a^ | |  |  |  | -2.10 (1.05) | -0.10 | -1.10 (1.05) | -0.05 | -1.14 (1.04) | -0.05 | -1.41 (1.05) | -0.06 |
| Maternal age^a^ |  |  |  |  | -0.13 (0.10) | -0.06 | -0.06 (0.10) | -0.03 | -0.09 (0.10) | -0.04 | -0.07 (0.10) | -0.03 |
| Maternal BMI^b^ |  |  |  |  | 1.04 (0.09) | 0.02 | 0.06 (0.09) | 0.03 | 0.09 (0.09) | 0.04 | 0.06 (0.09) | 0.03 |
| Reward for behaviour |  |  |  |  |  |  | 1.70 (0.82) | 0.11* | 1.66 (0.82) | 0.10* | 1.63 (0.85) | 0.10 |
| Reward for eating |  |  |  |  |  |  | 1.77 (0.76) | 0.12* | 1.73 (0.76) | 0.12* | 1.77 (0.77) | 0.12* |
| Covert restriction |  |  |  |  |  |  | -2.13 (0.54) | -0.17*** | -2.09 (0.54) | -0.17*** | -2.00 (0.54) | -0.16*** |
| Overt restriction |  |  |  |  |  |  | -0.34 (0.54) | -0.03 | -0.35 (0.54) | -0.03 | -0.49 (0.56) | -0.04 |
| Same food |  |  |  |  |  |  | 0.15 (0.40) | 0.02 | 0.14 (0.40) | 0.02 | 0.06 (0.44) | 0.01 |
| Group allocation (ref: NOURISH control/SAIDI) | | |  |  |  |  | -1.01 (1.03) | -0.04 | -1.13 (1.03) | -0.05 | -1.30 (1.04) | -0.06 |
| Child gender (ref: male) |  |  |  |  |  |  |  |  | 1.15 (0.91) | 0.05 | 1.10 (0.92) | 0.05 |
| Child age |  |  |  |  |  |  |  |  | -0.02 (8.26) | 0.00 | -1.76 (8.29) | -0.01 |
| Child BMI Z-score |  |  |  |  |  |  |  |  | -0.89 (0.46) | -0.08 | -0.72 (0.47) | -0.07 |
| Food responsiveness |  |  |  |  |  |  |  |  |  |  | -0.38 (0.96) | -0.02 |
| Enjoyment of food |  |  |  |  |  |  |  |  |  |  | 0.09 (1.14) | 0.01 |
| Satiety & slowness |  |  |  |  |  |  |  |  |  |  | 2.47 (1.11) | 0.12* |
| Food fussiness |  |  |  |  |  |  |  |  |  |  | -0.24 (0.93) | -0.02 |
| Emotional overeating |  |  |  |  |  |  |  |  |  |  | 1.75 (1.13) | 0.08 |
| Emotional undereating |  |  |  |  |  |  |  |  |  |  | -1.11 (0.58) | -0.09 |
| Desire to drink |  |  |  |  |  |  |  |  |  |  | -0.16 (0.58) | -0.01 |
| **Adjusted R^2^** | **0.025**** | | **0.034**** | | **0.040**** | | **0.108***** | | **0.112***** | | **0.117***** | |
| **R^2^ change** | **0.038**** | | **0.014** | | **0.011** | | **0.076***** | | **0.010** | | **0.016** | |

a At recruitment/child birth

b At Time 1/child age 4-6 months

ref = reference category * p<0.05; **p<0.01; ***p<0.001

Supplementary Table 2: Regression analyses of parental work hours and household income, family, parent and child factors, and proportion of total energy intake from discretionary foods and beverages (main meals and snacks combined) in 2-year-old Australian children (n=526)

|  | **Step 1** | | **Step 2** | | **Step 3** | | **Step 4** | | **Step 5** | | **Step 6** | |
| --- | --- | --- | --- | --- | --- | --- | --- | --- | --- | --- | --- | --- |
|  | **B (SE)** | **β** | **B (SE)** | **β** | **B (SE)** | **β** | **B (SE)** | **β** | **B (SE)** | **β** | **B (SE)** | **β** |
| Maternal working hrs (ref: not working) | |  |  |  |  |  |  |  |  |  |  |  |
| 1 to <21 hrs | -0.22 (0.87) | -0.01 | -0.15 (0.87) | -0.01 | 0.08 (0.88) | 0.00 | 0.01 (0.86) | 0.00 | 0.09 (0.86) | 0.01 | 0.19 (0.86) | 0.01 |
| 21 to <35 hrs | 1.43 (0.95) | 0.07 | 1.98 (0.98) | 0.10* | 2.28 (0.99) | 0.12* | 1.58 (0.97) | 0.08 | 1.84 (0.98) | 0.09 | 2.04 (0.98) | 0.10* |
| 35+ hrs | 0.12 (1.32) | 0.004 | 0.43 (1.34) | 0.02 | 0.61 (1.35) | 0.02 | -0.41 (1.33) | -0.02 | -0.27 (1.33) | -0.01 | -0.16 (1.33) | -0.01 |
| Paternal working hrs (ref: 35 to 40 hrs) | |  |  |  |  |  |  |  |  |  |  |  |
| Not working | -0.65 (1.91) | -0.02 | -0.02 (1.91) | 0.00 | 0.20 (1.92) | 0.01 | 1.02 (1.88) | 0.02 | 0.60 | 0.01 | 0.69 (1.90) | 0.02 |
| 1 to <35 hrs | -1.01 (1.23) | -0.04 | -1.54 (1.23) | -0.06 | -1.31 (1.23) | -0.05 | -0.87 (1.21) | -0.03 | -1.06 (1.21) | -0.04 | -1.12 (1.21) | -0.04 |
| >40 hrs | -1.43 (0.84) | -0.08 | -1.74 (0.83) | -0.09* | -1.79 (0.83) | -0.10* | -1.96 (0.81) | -0.11** | -1.96 (0.81) | -0.11* | -2.11 (0.81) | -0.11* |
| Household income (ref: <50k) | -3.15 (1.06) | -0.14** | -2.83 (1.13) | -0.12* | -2.38 (1.14) | -0.10* | -2.49 (1.12) | -0.11** | -2.45 (1.11) | -0.11* | -2.69 (1.12) | -0.12* |
| Partnered (ref: single) |  |  | 2.48 (2.42) | 0.05 | 2.37 (2.42) | 0.05 | 2.23 (2.36) | 0.04 | 2.50 (2.36) | 0.05 | 3.33 (2.39) | 0.06 |
| Paternal education (ref: no uni)^a^ |  |  | -2.74 (0.72) | -0.17*** | -2.03 (0.78) | -0.12* | -1.98 (0.77) | -0.12** | -1.90 (0.77) | -0.12* | -1.93 (0.77) | -0.12* |
| No of children (ref: one child) |  |  | 1.33 (0.76) | 0.08 | 1.46 (0.79) | 0.09 | 1.09 (0.78) | 0.07 | 1.05 (0.78) | 0.06 | 1.22 (0.79) | 0.07 |
| Maternal education (ref: no uni)^a^ | |  |  |  | -1.50 (0.80) | -0.09 | -0.88 (0.80) | -0.05 | -0.91 (0.76) | -0.05 | -1.10 (0.80) | -0.07 |
| Maternal age^a^ |  |  |  |  | -0.08 (0.08) | -0.05 | -0.05 (0.08) | -0.03 | -0.07 (0.08) | -0.04 | -0.06 (0.08) | -0.03 |
| Maternal BMI^b^ |  |  |  |  | 0.04 (0.07) | 0.02 | 0.05 (0.07) | 0.03 | 0.08 (0.07) | 0.05 | 0.06 (0.07) | 0.04 |
| Reward for behaviour |  |  |  |  |  |  | 0.82 (0.62) | 0.07 | 0.79 (0.62) | 0.07 | 0.82 (0.65) | 0.07 |
| Reward for eating |  |  |  |  |  |  | 1.25 (0.58) | 0.11** | 1.20 (0.58) | 0.11* | 1.35 (0.58) | 0.12* |
| Covert restriction |  |  |  |  |  |  | -1.44 (0.41) | -0.15** | -1.41 (0.41) | -0.15** | -1.33 (0.41) | -0.14** |
| Overt restriction |  |  |  |  |  |  | -0.30 (0.41) | -0.03 | -0.28 (0.41) | -0.03 | -0.31 (0.43) | -0.03 |
| Same food |  |  |  |  |  |  | -0.22 (0.30) | -0.03 | -0.20 (0.30) | -0.03 | -0.41 (0.33) | -0.06 |
| Group allocation (ref: NOURISH control/SAIDI) | | |  |  |  |  | -0.80 (0.78) | -0.05 | -0.94 (0.78) | -0.05 | -1.03 (0.79) | -0.06 |
| Child gender (ref: male) |  |  |  |  |  |  |  |  | 0.58 (0.69) | 0.04 | 0.40 (0.70) | 0.02 |
| Child age |  |  |  |  |  |  |  |  | 3.04 (6.28) | 0.02 | 1.57 (6.30) | 0.01 |
| Child BMI Z-score |  |  |  |  |  |  |  |  | -0.79 (0.35) | -0.10* | -0.70 (0.36) | -0.09 |
| Food responsiveness |  |  |  |  |  |  |  |  |  |  | -0.14 (0.73) | -0.01 |
| Enjoyment of food |  |  |  |  |  |  |  |  |  |  | 0.18 (0.86) | 0.01 |
| Satiety & slowness |  |  |  |  |  |  |  |  |  |  | 1.82 (0.84) | 0.12* |
| Food fussiness |  |  |  |  |  |  |  |  |  |  | -0.70 (0.70) | -0.06 |
| Emotional overeating |  |  |  |  |  |  |  |  |  |  | 0.99 (0.86) | 0.06 |
| Emotional undereating |  |  |  |  |  |  |  |  |  |  | -1.01 (0.44) | -0.11* |
| Desire to drink |  |  |  |  |  |  |  |  |  |  | -0.17 (0.44) | -0.02 |
| **Adjusted R^2^** | **0.018*** | | **0.050***** | | **0.055***** | | **0.104***** | | **0.110***** | | **0.114***** | |
| **R^2^ change** | **0.031*** | | **0.037***** | | **0.010** | | **0.058***** | | **0.011** | | **0.016***** | |

a At recruitment/child birth

b At Time 1/child age 4-6 months

ref = reference category * p<0.05; **p<0.01; ***p<0.001

Supplementary Table 3: Regression analyses of parental work hours and household income, family, parent and child factors, and proportion of total energy intake from discretionary foods and beverages (main meals and snacks combined) in 2-year-old Australian children (n=526)

|  | **Step 1** | | | **Step 2** | | **Step 3** | | **Step 4** | | | | **Step 5** | | **Step 6** | |
| --- | --- | --- | --- | --- | --- | --- | --- | --- | --- | --- | --- | --- | --- | --- | --- |
|  | **B (SE)** | **β** | **B (SE)** | | **β** | **B (SE)** | **β** | **B (SE)** | | **β** | | **B (SE)** | **β** | **B (SE)** | **β** |
| Maternal working hrs (ref: not working) | |  |  | |  |  |  |  |  | |  | |  |  |  |
| 1 to <21 hrs | 0.66 (0.64) | 0.05 | 0.77 (0.65) | | 0.06 | 0.91 (0.65) | 0.07 | 0.83 (0.65) | | 0.06 | | 0.85 (0.65) | 0.06 | 0.83 (0.65) | 0.06 |
| 21 to <35 hrs | 0.58 (0.69) | 0.04 | 0.68 (0.72) | | 0.05 | 0.87 (0.73) | 0.06 | 0.59 (0.73) | | 0.04 | | 0.68 (0.74) | 0.05 | 0.67 (0.74) | 0.05 |
| 35+ hrs | -0.57 (0.96) | -0.03 | -0.38 (0.99) | | -0.02 | -0.25 (1.00) | -0.01 | -0.70 (1.00) | | -0.03 | | -0.60 (0.98) | -0.03 | -0.53 (1.00) | -0.03 |
| Paternal working hrs (ref: 35 to 40 hrs) | |  |  | |  |  |  |  |  | |  | |  |  |  |
| Not working | -3.44 (1.39) | -0.11* | -3.52 (1.42) | | -0.11* | -3.38 (1.42) | -0.11* | -2.84 (1.41) | | -0.09* | | -3.02 (1.42) | -0.10* | -2.84 (1.43) | -0.09* |
| 1 to <35 hrs | -0.15 (0.90) | -0.01 | -0.15 (0.91) | | -0.01 | -0.03 (0.91) | -0.00 | 0.09 (0.91) | | 0.00 | | 0.04 (0.91) | 0.00 | -0.01 (0.91) | -0.00 |
| >40 hrs | -0.33 (0.61) | -0.03 | -0.28 (0.62) | | -0.02 | -0.31 (0.62) | -0.02 | -0.31 (0.61) | | -0.02 | | -0.33 (0.61) | -0.03 | -0.26 (0.61) | -0.02 |
| Household income (ref: <50k) | -2.18 (0.77) | -0.13* | -2.40 (0.83) | | -0.14** | -2.14 (0.85) | -0.13* | -2.14 (0.84) | | -0.13* | | -2.10 (0.84) | -0.13* | -2.16 (0.84) | -0.13* |
| Partnered (ref: single) |  |  | -0.14 (1.80) | | -0.00 | -0.18 (1.80) | -0.01 | -0.21 (1.77) | | -0.01 | | -0.08 (1.77) | -0.00 | -0.00 (1.79) | 0.00 |
| Paternal education (ref: no uni)^a^ | |  | 0.83 (0.54) | | 0.07 | 1.23 (0.58) | 0.10* | 1.37 (0.57) | 0.11* | | 1.41 (0.58) | | 0.12* | 1.44 (0.58) | 0.12* |
| No of children (ref: one child) |  |  | 0.26 (0.56) | | 0.02 | 0.38 (0.59) | 0.03 | 0.08 (0.59) | | 0.01 | | 0.04 (0.59) | 0.00 | 0.08 (0.59) | 0.01 |
| Maternal education (ref: no uni)^a^ | |  |  | |  | -0.81 (0.59) | -0.07 | -0.30 (0.60) | -0.02 | | -0.32 (0.60) | | -0.03 | -0.41 (0.60) | -0.03 |
| Maternal age^a^ |  |  |  | |  | -0.06 (0.06) | -0.05 | -0.03 (0.06) | | -0.02 | | -0.04 (0.06) | -0.03 | -0.04 (0.06) | -0.03 |
| Maternal BMI^b^ |  |  |  | |  | 0.01 (0.05) | 0.01 | 0.02 (0.05) | | 0.02 | | 0.03 (0.05) | 0.03 | 0.02 (0.05) | 0.02 |
| Reward for behaviour |  |  |  | |  |  |  | 0.85 (0.47) | | 0.10 | | 0.83 (0.47) | 0.09 | 0.73 (0.49) | 0.08 |
| Reward for eating |  |  |  | |  |  |  | 0.43 (0.43) | | 0.05 | | 0.44 (0.43) | 0.06 | 0.28 (0.44) | 0.04 |
| Covert restriction |  |  |  | |  |  |  | -0.94 (0.31) | | -0.14** | | -0.92 (0.31) | -0.14** | -0.94 (0.31) | -0.14** |
| Overt restriction |  |  |  | |  |  |  | -0.17 (0.31) | | -0.03 | | -0.18 (0.31) | -0.03 | -0.34 (0.32) | -0.05 |
| Same food |  |  |  | |  |  |  | 0.28 (0.23) | | 0.06 | | 0.26 (0.23) | 0.05 | 0.44 (0.25) | 0.09 |
| Group allocation (ref: NOURISH control/SAIDI) | | | |  |  |  |  | -0.72 (0.59) | | -0.06 | | -0.72 (0.59) | -0.06 | -0.81 (0.59) | -0.06 |
| Child gender (ref: male) |  |  |  | |  |  |  |  | |  | | 0.51 (0.52) | 0.04 | 0.68 (0.53) | 0.06 |
| Child age |  |  |  | |  |  |  |  | |  | | -3.25 (4.72) | -0.03 | -3.21 (4.74) | -0.03 |
| Child BMI Z-score |  |  |  | |  |  |  |  | |  | | -0.28 (0.26) | -0.05 | -0.18 (0.27) | -0.03 |
| Food responsiveness |  |  |  | |  |  |  |  | |  | |  |  | -0.20 (0.55) | -0.02 |
| Enjoyment of food |  |  |  | |  |  |  |  | |  | |  |  | 0.30 (0.65) | 0.03 |
| Satiety & slowness |  |  |  | |  |  |  |  | |  | |  |  | 0.67 (0.63) | 0.06 |
| Food fussiness |  |  |  | |  |  |  |  | |  | |  |  | 0.81 (0.53) | 0.09 |
| Emotional overeating |  |  |  | |  |  |  |  | |  | |  |  | 0.68 (0.65) | 0.06 |
| Emotional undereating |  |  |  | |  |  |  |  | |  | |  |  | 0.16 (0.34) | 0.02 |
| Desire to drink |  |  |  | |  |  |  |  | |  | |  |  | -0.03 (0.33) | -0.00 |
| **Adjusted R^2^** | **0.016*** | | **0.015** | | | **0.016** | | **0.049**** | | | | **0.049**** | | **0.052**** | |
| **R^2^ change** | **0.029*** | | **0.005** | | | **0.006** | | **0.044**** | | | | **0.005** | | **0.016**** | |

a At recruitment/child birth

b At Time 1/child age 4-6 months

ref = reference category * p<0.05; **p<0.01; ***p<0.001
